# Serum type III procollagen, procollagen V, tumour necrosis factor-a, interleukin-6 in liver cirrhosis after antiviral treatment

**DOI:** 10.5937/jomb0-56353

**Published:** 2025-10-28

**Authors:** Wei Li, Xueshuang Liu, Fan Bi

**Affiliations:** 1 Dazhou Vocational College of Chinese Medicine, Department of Basic Medical Education, Dazhou, 635000, Sichuan Province, China

**Keywords:** serum type III procollagen (PCIII), plasma procollagen V (IVC), tumour necrosis factor-a (TNF-a), interleukin-6 (IL-6), entecavir, reduced glutathione, alcoholic cirrhosis, hepatitis B, clinical effects, nutritional status, serumski prokolagen tipa III (PCIII), plazmatski prokolagen V (IVC), faktor nekroze tumora-a (TNF-a), interleukin-6 (IL-6), entekavir, redukovani glutation, alkoholna ciroza, hepatitis B, klinički efekti, nutritivni status

## Abstract

**Background:**

To evaluate the therapeutic effects of entecavir combined with reduced glutathione on serum type III procollagen (PCIII), plasma procollagen V (IVC), Tumour Necrosis Factor-a (TNF-a), interleukin-6 (IL-6), and nutritional status in patients with hepatitis B complicated by alcoholic liver cirrhosis.

**Methods:**

This study included 92 patients with alcoholic liver cirrhosis and hepatitis B, treated between April 2022 and January 2024. Patients were randomised into two groups: group A received 0.5 mg of entecavir daily, and group B received 0.5 mg of entecavir daily plus 0.3 g of reduced glutathione 1-2 times per day for 2 months. Nutritional parameters, inflammatory markers, liver function, and malnutrition were compared between groups. Statistical analysis was performed using SPSS12.0, with independent t-tests for group comparisons and x2 tests for categorical data. A P-value &lt;0.05 was considered statistically significant.

**Results:**

Group B showed a higher effective treatment rate (97.82%) compared to group A (76.08%). Malnutrition improved significantly more in group B. After treatment, group B exhibited more significant reductions in BMI, TSF, AMC, PA, Hb, ALB, TNF-a, CRP, IL-6, TBIL, AST, ALT, HA, PCIII, and IVC than group A.

**Conclusions:**

Entecavir combined with reduced glutathione improves liver function and nutritional status and reduces inflammatory markers in patients with hepatitis B and alcoholic liver cirrhosis, demonstrating high safety and effectiveness.

## Abstract (sr)

**Uvod:**

Procena terapijskih efekata entekavira u kombinaciji sa redukovanim glutationom na serumski prokolagen tipa III (PCIII), plazmatski prokolagen V (IVC), faktor nekroze tumora-a (TNF-a), interleukin-6 (IL-6) i nutritivni status kod pacijenata sa hepatitisom B komplikovanom alkoholnom cirozom jetre.

**Metode:**

U studiju je uključeno 92 pacijenta sa alkoholnom cirozom jetre i hepatitisom B, lečenih u periodu od aprila 2022. do januara 2024. Pacijenti su nasumično raspoređeni u dve grupe: grupa A je primala 0,5 mg entekavira dnevno, dok je grupa B primala 0,5 mg entekavira dnevno uz 0,3 g redukovanog glutationa 1-2 puta dnevno tokom 2 meseca. Upoređeni su nutritivni parametri, inflamatorni markeri, funkcija jetre i stanje neuhranjenosti između grupa. Statistička analiza je sprovedena korišćenjem SPSS 12.0, pri čemu su za poređenje grupa korišćeni nezavisni t-testovi, a za kategorijske podatke hi-kvadrat test (x2). P-vrednost &lt;0,05 smatrana je statistički značajnom.

**Rezultati:**

Grupa B je imala višu stopu efikasnog lečenja (97,82%) u poređenju sa grupom A (76,08%). Neuhra njost se značajno više poboljšala u grupi B. Nakon terapije, grupa B je pokazala veće smanjenje BMI, TSF, AMC, PA, Hb, ALB, TNF-a, CRP, IL-6, TBIL, AST, ALT, HA, PCIII i IVC u odnosu na grupu A.

**Zaključak:**

Entekavir u kombinaciji sa redukovanim glutationom poboljšava funkciju jetre, nutritivni status i smanjuje inflamatorne markere kod pacijenata sa hepatitisom B i alkoholnom cirozom jetre, pokazujući visoku bezbednost i efikasnost.

## Introduction

Hepatitis B (HB) is a chronic viral infection that primarily affects the liver, leading to inflammation and potential long-term damage. If untreated, HB can progress to cirrhosis, liver failure, or even liver cancer [1]. Alcoholic liver cirrhosis, which results from prolonged and excessive alcohol consumption, is characterised by severe liver fibrosis and scarring [2]. When both conditions co-occur, the liver is under increased stress, leading to more severe liver dysfunction and a higher risk of complications such as malnutrition, fatigue, and decreased life expectancy [3]. Therefore, managing both hepatitis B and alcoholic liver cirrhosis requires a multifaceted approach to improve liver function, control inflammation, and address nutritional deficiencies.

Entecavir is an effective antiviral agent that suppresses hepatitis B virus (HBV) replication and reduces liver inflammation, making it a cornerstone of Hb treatment [4]. However, for patients with both Hb and alcoholic cirrhosis, antiviral therapy alone may not be sufficient to fully restore liver function, reduce fibrosis, or address the complex nutritional issues that often arise from liver dysfunction. In these patients, liver injury is compounded by oxidative stress and inflammation, which not only exacerbates liver damage but also impairs nutritional absorption and metabolism [5].

Reduced glutathione, a potent antioxidant, plays a vital role in reducing oxidative stress and protecting liver cells from further damage. By scavenging free radicals and aiding in the repair of damaged liver cells, glutathione helps mitigate the detrimental effects of both HBV infection and alcohol-induced liver injury [6]. Given its protective role, some researchers have proposed that combining entecavir with reduced glutathione may offer a synergistic effect – entecavir suppresses viral replication while glutathione promotes liver regeneration and reduces oxidative damage. This combination could theoretically improve liver function, reduce inflammation, and support nutritional recovery in patients with both hepatitis B and alcoholic cirrhosis.

Despite the potential benefits of this combined approach, there is a gap in the existing literature regarding its impact on the nutritional status and overall quality of life in these patients. While much of the research has focused on the individual benefits of entecavir and glutathione, few studies have evaluated how their combination affects not only liver function but also nutritional outcomes. Malnutrition is a common issue in patients with cirrhosis, leading to poor prognosis and decreased quality of life. Still, this aspect has been largely overlooked in clinical trials evaluating antiviral or antioxidant therapies. Therefore, this study aims to address this gap by assessing the clinical effects of entecavir combined with reduced glutathione in patients with hepatitis B complicated by alcoholic liver cirrhosis, with a specific focus on its impact on nutritional status and overall health outcomes.

## Materials and methods

### General information

Ninety-two patients with hepatitis B complicated with alcoholic cirrhosis who received treatment in our hospital from April 2022 to January 2024 were selected as the research objects, and the patients were divided into a control group (group A) (n=46) and an experimental group (group B) (n=46). Gender, age, illness stage, and drinking history did not differ significantly between the two groups (P>0.05) (Table 1).

**Table 1:** General information [n/% (x̄±s)].

Group	N	Gender<br>(male/ <br>female)	Age <br>(years)	Disease <br>duration <br>(years)	Drinking <br>history <br>(years)
A	46	24/22	52.36±2.27	5.11±1.26	3.62±1.13
B	46	23/23	52.47±2.36	5.19±1.21	3.71±1.34
* x^2^/t *		0.043	0.227	0.311	0.348
* p *		0.834	0.820	0.756	0.728

### Inclusion and exclusion criteria

Criteria for inclusion: ① fulfil the diagnostic requirements for individuals with alcoholic cirrhosis and severe hepatitis B (Liberal and Grant, 2016); ② The age is 18–75 years old, positive for five hepatitis B surface antigens, HBVDNA>104·mL-1, portal vein inner diameter 1.3 cm, spleen thickness >4.0 cm, total bilirubin >34.20 μmol·L-1, abnormal liver function; ③ A long-term history of drinking, accompanied by jaundice and right upper abdominal pain; ④ The patient voluntarily participated in clinical treatment and signed the informed consent form.

Exclusion criteria: ① Liver cirrhosis caused by drugs, autoimmune, and metabolic diseases; ② Pregnant and nursing patients and patients with a history of allergies or intolerance to entecavir combined with reduced glutathione; ③ Patients with cardiovascular and renal dysfunction and malignant tumours; ④ Other severe comorbidities or mental disorders that affect treatment implementation and follow-up.

### Treatment

Patients in both groups were given nutritional support, anti-infection, and correction of acid-base balance disorders. On this basis, the control group was given entecavir (Manufacturer: Haisike Pharma ceutical (Meishan) Co., Ltd., Chinese Medicine Approval: H20130031, specification: 0.5 mg*7 capsules) 0.5 mg/day treatment; the experimental group was treated with reduced glutathione based on the control group (Manufacturer: Qingdao Jieshikang Biotechnology Co., Ltd., Chinese Medicine Approval: H20130135, specification: 0.1 g/bottle) 5% glucose injection diluted with 0.3 g/1–2 times/d, reduced glutathione was injected intravenously for treatment. Patients in both groups were treated continuously for 2 months.

### Outcome measures

(1) Examine the differences in the two patient groups’ clinical response to therapy. Effective treatment: TBIL decreased by more than 50%, PTA increased by more than 10%, HBVDNA turned negative, and signs such as jaundice, ascites, and right upper abdominal pain disappeared or significantly reduced. Effective treatment: TBIL decreased by more than 30%, PTA increased slightly, HBVDNA decreased by at least two orders of magnitude, and signs such as jaundice, ascites, and right upper abdominal pain were alleviated. Ineffective treatment: The improvement criterion was not reached. Total effective rate = (effective + effective)/total number of cases ×100%.

(2) Compare the body index of patients in the two groups [BMI=weight/(height) 2], triple scalp thickness (TSF) from the neck to the midpoint of the skeletal muscles skin thickness at 2 centimetres upward, superior arm (AMC), AMC= AMC-pixTSF (cm) [7].

(3) Child-Pugh grading standard for liver reserve function assessment of liver cirrhosis: Grade A has good liver function (5–6 points), Grade B has moderate liver function (7–9 points), and Grade C has poor liver function ( 10 points); Nutritional risk screening using the Subjective Comprehensive Evaluation (SGA) includes dietary intake, weight change, gastrointestinal symptoms, daily function, disease stress and physical examination. The scoring levels are divided into 3 levels. Level A is no malnutrition, Level B is mild to moderate malnutrition, and Level C is severe malnutrition.

(4) Before and two months after therapy, 5 mL of elbow vein blood was drawn from patients in the two groups while they were fasting. After centrifugation, the supernatant was placed in a -80°C environment to detect inflammatory factors [Tumour Necrosis Factor-α] before and after treatment. (Tumour necrosis factor-α, TNF-α), high-sensitivity C-reactive protein (CRP), interleukin-6 (Interleukin-6, IL-6)] expression levels were detected by ELISA (Manufacturer: ThermoFisher Scientific), liver function indicators [total bilirubin (TBIL), serum aspartate aminotransferase (AST), alanine aminotransferase (ALT)], serum protein [serum prealbumin (PA), haemoglobin (Hb), albumin (ALB)] were detected using a fully automatic biochemical analyser (Manufacturer: Roche Diagnostics), liver fibrosis indicators [dialysis heparin (HA), type IIIprocollagen (PCIII), plasma procollagen V (IVC)] were detected using immunoradiology (manufacturer: Merck Millipore).

(5) Compare the incidence of adverse reactions such as abdominal pain, dizziness, fever, and liver discomfort between the two groups.

### Statistical methods

Statistical analysis was done using the SPSS 12.0 program. The measured data was presented as mean±standard deviation (x̄±s). The independent sample t-test was employed to compare groups. Percentages (%) were used to convey counting data. The x^2^ test was applied. Since the difference was statistically significant, P<0.05 was chosen.

## Results

### The clinical treatment effects

Compared to group A (76.08%), group B’s overall effective rate was higher at 97.82%. See Table 2 for details.

**Table 2:** The clinical treatment effects (n/%).

Group	N	markedly<br>effective <br>(%)	Effective <br>(%)	Invalid <br>(%)	Total <br>effective <br>rate (%)
A	46	24(52.16)	11(23.92)	11(23.92)	35(76.08)
B	46	36(78.26)	9(19.56)	1(2.18)	45(97.82)
χ^2^					36.507
* p *					<0.001

### The level of BMI, TSF, AMC, PA, Hb, MCB

Prior to therapy, the two groups’ Body Mass Index (BMI), Triceps Skinfold Thickness (TSF), Arm Muscle Circumference (AMC), Prealbumin (PA), Hemoglobin (Hb), and Albumin (ALB) did not differ statistically significantly. Compared with before treatment, after treatment, BMI, TSF, AMC, PA, Hb, and ALB in the two groups increased, and group B was better than group A (Table 3).

**Table 3:** The level of BMI, TSF, AMC, PA, Hb, MCB (x̄±s).

Group	N	BMI				TSF (mm)			
		before<br>treatment	after <br>treatment	t	p	before <br>treatment	after <br>treatment	* t *	*p *
A	46	16.25±1.68	17.26±1.35	3.178	<0.001	8.17±1.35	10.52±4.23	3.589	<0.001
B	46	16.37±1.24	19.27±2.52	7.003	<0.001	8.24±1.22	15.26±4.69	9.824	<0.001
* t *		0.389	4.768			0.261	5.09		
* p *		0.697	<0.001			0.794	<0.001		
Group	N	AMC (cm)				PA (mg/L)			
		before <br>treatment	after <br>treatment	t	p	before <br>treatment	after <br>treatment	* t *	* p *
A	46	18.35±2.13	20.24±5.06	2.334	<0.001	67.52±4.33	71.29±9.37	2.477	0.015
B	46	18.65±2.09	23.49±5.26	5.799	<0.001	67.82±4.21	86.37±9.69	11.908	<0.001
* t *		0.681	3.02			0.336	7.587		
* p *		0.497	<0.001			0.737	<0.001		
Group	N	Hb g/L				ALB g/L			
		before <br>treatment	after <br>treatment	t	p	before <br>treatment	after <br>treatment	* t *	* p *
A	46	86.61±8.22	91.31±13.19	2.051	0.043	24.31±3.14	26.81±5.01	2.867	0.005
B	46	86.25±11.62	103.26±21.62	4.423	<0.001	24.56±3.25	31.27±5.23	7.391	<0.001
* t *		0.171	3.2			0.375	4.176		
* p *		0.864	0.019			0.708	<0.001		

### Compare the incidence of malnutrition and SGA

Prior to treatment, there was no statistically significant variation in the incidence of malnutrition between the two groups. After treatment, compared with before treatment, the level of malnutrition was reduced, and group B was better than group A (see Table 4 for details).

**Table 4:** Compare the incidence of malnutrition and SGA (n/%).

Child pugh			N	SGA				x^2^	* p *
				Normal	A-level	B-level	C-level		
before<br>treatment	A	A-level	4	2(50.0)	1(25.0)	1(25.0)	0(0)	11.181	0.082
		B-level	36	28(77.78)	1(2.78)	6(6.67)	1(2.78)		
		C-level	6	2(33.33)	2(33.33)	1(16.67)	1(16.67)		
		** Total **	** 46 **	** 32(69.56) **	** 4(8.69) **	** 8(17.39) **	** 2(4.34) **		
	B	A-level	4	2(50.0)	1(25.0)	1(25.0)	0(0)	6.983	0.322
		B-level	36	25(72.22)	2(5.55)	7(16.68)	2(5.55)		
		C-level	6	2(33.33)	2(33.33)	1(16.67)	1(16.67)		
		** Total **	** 46 **	** 29(63.04) **	** 5(10.86) **	** 9(19.56) **	** 3(6.52) **		
after<br>treatment	A	A-level	4	3(75.00)	1(25.00)	0	0	4.765	0.574
		B-level	36	28(77.78)	3(8.33)	4(11.11)	1(2.78)		
		C-level	6	3(50.0)	1(16.67)	1(16.67)	1(16.67)		
		** Total **	** 46 **	** 34(73.91) **	** 5(10.86) **	** 5(10.86) **	** 2(4.34) **		
	B	A-level	4	4(100.00)	0	0	0	1.145	<0.001
		B-level	36	32(83.33)	3(8.33)	1(2.77)	0		
		C-level	6	5(83.33)	1(16.67)	0	0		
		** Total **	** 46 **	** 41(89.13) **	** 4(8.69) **	** 1(2.17) **	** 0 **		

### The expression levels of serum inflammatory factors

Prior to therapy, there was no discernible variation in TNF-α, CRP, or IL-6 expression levels between the two groups. Following therapy, both groups’ TNF-α, CRP, and IL-6 expression levels dropped from pre-treatment levels, with group B’s levels being lower than those of group A (Table 5).

**Table 5:** The expression levels of serum inflammatory factors (x̄±s).

Group	N	TNF-α				CRP (mg/L)			
		before<br>treatment	after<br>treatment	* t *	* p *	before<br>treatment	after<br>treatment	* t *	* p *
A	46	16.39±1.25	15.33±1.25	4.066	<0.001	425.33±20.26	75.39±12.38	99.962	<0.001
B	46	16.84±1.84	11.25±1.16	17.43	<0.001	425.29±20.65	50.26±10.12	110.607	<0.001
* t *		1.372	16.226			0.009	10.659		
* p *		0.173	<0.001			0.992	<0.001		
Group	N	IL-6 (pg/mL)							
		before<br>treatment	after<br>treatment	* t *	* p *				
A	46	257.52±24.29	144.29±24.96	22.05	<0.001				
B	46	258.10±23.36	117.36±15.47	34.068	<0.001				
* t *		0.116	6.219						
* p *		0.907	<0.001						

### The expression levels of liver function indicators TBIL, AST and ALT

Before therapy, there was no statistically significant difference in the two groups’ TBIL, AST, and ALT expression levels. Following treatment, both groups’ TBIL, AST, and ALT expression levels dropped from their pre-treatment values; group B’s expression levels were lower than those of group A (Table 6).

**Table 6:** The expression levels of liver function indicators TBIL, AST and ALT (x̄±s).

Group	N	TBIL (μmol/L)				AST (U/L)			
		before<br>treatment	after<br>treatment	*t *	* p *	before<br>treatment	after<br>treatment	* t *	* p *
A	46	54.27±15.33	34.26±8.33	7.778	<0.001	134.69±22.25	69.36±13.25	17.11	<0.001
B	46	54.46±15.28	26.75±8.36	10.79	<0.001	134.86±22.67	51.25±11.56	22.284	<0.001
* t *		0.059	4.316			0.036	6.985		
* p *		0.952	<0.001			0.971	<0.001		
Group	N	ALT(U/L)							
		before<br>treatment	after<br>treatment	* t *	* p *				
A	46	144.35±25.26	117.25±14.36	6.325	<0.001				
B	46	144.21±24.35	94.36±13.25	12.196	<0.001				
* t *		0.027	7.945						
* p *		0.978	<0.001						

### The expression levels of liver fibrosis indicators HA, PCIII and IVC

Before treatment, there was no statistically significant difference in the expression levels of HA, PCIII, and IVC between the two groups. Following therapy, both groups’ levels of HA, PCIII, and IVC expression were lower than they were prior to treatment, with group B’s levels being lower than those of group A (Table 7).

**Table 7:** The expression levels of liver fibrosis indicators HA, PCIII and IVC(x̄±s).

Group	N	HA (mg/L)				PCIII (μg/L)			
		before<br>treatment	after<br>treatment	t	p	before<br>treatment	after<br>treatment	* t *	* p *
A	46	203.25±15.45	155.37±14.28	15.435	<0.001	203.25±16.52	163.38±14.26	20.83	<0.001
B	46	202.96±15.28	112.29±11.41		<0.001	203.16±16.33	107.52±11.29	32.673	<0.001
* t *		0.091	15.985			0.026	20.83		
*p*		0.928	<0.001			0.979	<0.001		
Group	N	IVC (μg/L)							
		before<br>treatment	after<br>treatment	* t *	* p *				
A	46	165.22±16.33	133.29±12.27	10.602	<0.001				
B	46	165.41±16.29	111.28±10.36	19.017	<0.001				
* t *		0.055	9.295						
* p *		0.955	<0.001						

### The incidence of adverse reactions

The incidence of adverse responses (4.34%) in group B and 13.04% in group A did not vary statistically (Table 8).

**Table 8:** The incidence of adverse reactions (n/%).

Group	N	stomachache	swirl	high fever	Liver<br>discomfort	Overall incidence<br>of adverse reactions (%)
A	46	1(2.17)	2(4.34)	2(4.34)	1(2.17)	6(13.04)
B	46	0	1(2.17)	1(2.17)	0	2(4.34)
* t *						2.191
* p *						0.138

## Discussion

This study demonstrates that entecavir combined with reduced glutathione can significantly improve the nutritional status, liver function, and inflammation levels in patients with hepatitis B complicated by alcoholic cirrhosis. Group B, which received the combined therapy, showed better results compared to group A, with reduced malnutrition grades and lower levels of inflammatory markers such as TNF-α, CRP, and IL-6 after treatment. Furthermore, the study found a reduction in liver fibrosis markers, including HA, PCIII, and IVC, in group B, suggesting that the combination therapy may also contribute to the protection of liver tissue and improvement in liver function.

Cirrhosis is affected by many factors, including genetics, alcohol history and nutritional status [8]. A prevalent feature of end-stage liver disease is malnutrition, mainly due to insufficient intake and absorption dysfunction. Patients with end-stage liver disease often have symptoms such as loss of appetite, taste changes, nausea, vomiting, etc., resulting in reduced food intake. When patients have ascites, ascites press the gastrointestinal tract, resulting in early satiety. Treatment for ascites requires fasting, such as CT and gastroscopy, resulting in reduced nutrient intake. 35% to 60% of patients will have intestinal microecological changes, resulting in nutrient absorption disorders. Cholestasis reduces the total amount of bile in the intestinal cavity, causing steatosis and absorption disorders of fat-soluble vitamins. Antibiotics and other drugs can also affect intestinal function and further affect nutrient absorption. At present, the nutritional status of patients with cirrhosis of different causes has not been fully studied. Now, most researchers propose that with the change of lifestyle, more and more patients appear to have hepatitis B complicated with alcoholic cirrhosis. When hepatitis B infection occurs in China, alcoholic cirrhosis is mainly common, and the combination of the two will accelerate the process of cirrhosis. When hepatitis B complicated with alcoholic cirrhosis occurs, patients are more likely to develop decompensated cirrhosis complicated with liver failure, in which there are multiple risk factors interacting and aggravating the condition [9]. Major pathophysiological studies have found that alcohol can affect a variety of enzyme activities, resulting in metabolic disorders and decreased immune function [10]. Entecavir is an antiviral drug that effectively inhibits hepatitis B virus replication [11]. Reduced glutathione is an antioxidant that reduces free radical damage to cells and promotes the metabolism of toxic substances in the liver. Combined usage of the two can enhance survival rates, improve liver function, and lessen the condition of patients with decompensated Hb [12]. According to this study, group B fared better than group A, and the malnutrition grade decreased after therapy compared to before treatment. It can be seen that entecavir combined with reduced glutathione can improve the nutritional status of hepatitis B patients with alcoholic cirrhosis.

TNF-α is an inflammatory mediator produced by immune cells and, when abnormally expressed, promotes the development of hepatitis and liver fibrosis [13]. Wu et al. [14] found that reducing TNF-α promotes recovery from cirrhosis. CRP is an acute inflammatory marker, and its level will increase significantly in an inflammatory state, which can sense infection or injury site of the body in time [15]. IL-6 is a pro-inflammatory cytokine that can promote inflammatory response and the activation of immune cells. Wang et al. [16] found that IL-6 participates in the occurrence and progression of liver fibrosis in the development of liver diseases, affecting the regeneration ability of the liver. Abnormal expression of TNF-α, CRP and IL-6 inflammatory markers can promote liver inflammation, fibrosis and damage and aggravate liver function damage and disease severity of hepatitis B complicated with alcoholic cirrhosis. Therefore, monitoring and regulating the levels of these markers may be critical for managing the condition and prognosis of patients with Hb and alcoholic cirrhosis. The study’s findings indicate that there was no statistically significant difference in the levels of TNF-α, CRP, and IL-6 between the two groups prior to treatment. However, following treatment, the levels of these markers were lower in group B than in group A, indicating that entecavir, in conjunction with reduced glutathione treatment, may be able to lower these markers and improve the effectiveness of treatment for hepatitis B complicated by alcoholic cirrhosis.

TBIL is a processed form of bilirubin that may cause abnormalities in the biliary system, affecting bilirubin excretion and metabolism. Elevated TBIL may reflect hepatocellular injury and cholestasis and may help diagnose hepatocellular injury complicated by alcoholic cirrhosis [17]. AST and ALT mainly exist in liver cells [18]. When liver cells are damaged, AST will be released into the blood, which will increase AST, which can reflect the degree of liver cell damage and help to evaluate the condition of alcoholic cirrhosis patients [19]. Compared with AST, ALT has a higher sensitivity to liver cell injury and can be applied to the changes of early alcoholic cirrhosis patients. The findings demonstrated that both groups’ post-treatment HA, PCIII, and IVC expression levels were lower than those observed before the treatment, with group B’s levels being lower than those of group A. It is suggested that entecavir combined with reduced glutathione treatment can reduce the expression levels of HA, PCIII and IVC, improve the quality of life and promote the recovery of liver function.

HA is a glycoprotein that is usually released when the liver is damaged [20]. Studies have found that [21] liver damage may lead to an increase in HA levels in hepatitis B complicated with alcoholic cirrhosis. PCIII is a connective tissue protein. Karsdal et al. [22] have found that PCIII can be used as an essential indicator for predicting liver fibrosis and liver dysfunction in the early stage and can be used as a potential biological marker. IVC is the precursor of collagen, which can be used as the main component of the basement membrane. When its expression is abnormal, it indicates active liver fibrosis [5]. According to the study’s findings, both groups’ post-treatment HA, PCIII, and IVC expression levels were lower than they were pre-treatment, with group B’s levels being lower than those of group A. It is suggested that entecavir, combined with reduced glutathione treatment, can improve liver fibrosis and protect liver tissue.

This study suggests that entecavir combined with reduced glutathione may improve liver function, reduce inflammation, and enhance the nutritional status of patients with hepatitis B complicated by alcoholic cirrhosis. The potential mechanisms behind the synergistic effects of this combination therapy include the antiviral action of entecavir, which suppresses HBV replication, and the antioxidant properties of reduced glutathione, which helps reduce oxidative stress and protect liver cells from damage. Reduced glutathione may also play a role in modulating the immune response by lowering levels of inflammatory markers such as TNF-α, CRP, and IL-6, which are known to contribute to liver fibrosis and inflammation [13] [14] [16]. By reducing oxidative damage and inflammation, glutathione may create a more favourable environment for the antiviral effects of entecavir, leading to better overall liver function and potentially improving clinical outcomes.

This study has several limitations. The relatively short follow-up period of 2 months restricts the ability to assess the long-term effects of the combination therapy on liver function, nutritional status, and quality of life. Additionally, the lack of a placebo control group limits the ability to conclusively determine whether the observed improvements were solely due to the combination therapy or other factors. The study also did not account for potential confounding variables such as the patient’s alcohol consumption history, genetics, or other comorbidities, which may have influenced the outcomes. These factors highlight the need for further research with a longer follow-up period and a placebo-controlled design to confirm the efficacy of this treatment.

## Conclusion

Entecavir combined with reduced glutathione demonstrates potential in improving liver function, reducing inflammation, and enhancing nutritional status in patients with hepatitis B complicated by alcoholic cirrhosis. This combination therapy offers a promising approach for managing these patients, improving clinical outcomes, and delaying liver fibrosis progression. However, further research with a more extended follow-up period and placebo-controlled trials is needed to confirm the long-term benefits and evaluate the impact on patients’ quality of life.

## Dodatak

### List of abbreviations

BMI, body mass index;<br>TSF, triceps skinfold thickness;<br>AMC, arm muscle circumference; <br>PA, prealbumin;<br>HB, haemoglobin;<br>ALB, albumin;<br>TNF-α, tumour necrosis factor-α;<br>IL-6, interleukin-6;<br>CRP, C-reactive protein;<br>TBIL, total bilirubin;<br>AST, aspartate aminotransferase;<br>ALT, alanine aminotransferase;<br>HA, hyaluronic acid;<br>PCIII, procollagen type III;<br>IVC, procollagen type V;<br>HBV, hepatitis B virus;<br>SGA, subjective global assessment;<br>PA, proalbumin;<br>SPSS, statistical package for the social sciences.

### Conflict of interest statement

All the authors declare that they have no conflict of interest in this work.
